# An Effective Protocol for Micropropagation of Edible Bamboo Species (*Bambusa tulda* and *Melocanna baccifera*) through Nodal Culture

**DOI:** 10.1155/2014/345794

**Published:** 2014-05-21

**Authors:** Sayanika Devi Waikhom, Bengyella Louis

**Affiliations:** ^1^Institute of Bioresources and Sustainable Development, Takyelpat, Imphal, Manipur 795001, India; ^2^Centre of Advanced Study in Life Sciences, Manipur University, Imphal, Manipur 795003, India; ^3^Department of Biotechnology, The University of Burdwan, Golapbag More, West Bengal 713104, India; ^4^Department of Biochemistry, University of Yaoundé I, BP 812, Yaounde, Cameroon

## Abstract

High demand for edible bamboo shoots of *Bambusa tulda* and *Melocanna baccifera* in many Asian ethnic groups has led to the need for developing intensive bamboo farming. To achieve this, *in vitro* regeneration of bamboo plantlets is needed due to the long and irregular bamboo flowering cycle and scarcity of bamboo seeds. An effective protocol for plantlets regeneration in *B. tulda* and *M. baccifera* from nodal explants following validation of the species using the sequence of trnL-F intergenic spacer region is described. Effective axillary bud breaking was achieved at 3 mg/L of 6-benzylaminopurine (BAP) in MS medium. Importantly, combining 2 mg/L of kinetin (Kn) with 3 mg/L of BAP produced a synergistic effect for shoot multiplication in *B. tulda* and *M. baccifera*. Under optimized conditions in half-strength MS medium supplemented with 3 mg/L of indole-3-butyric acid (IBA), 10 mg/L of coumarin, and 3% sucrose, profuse production of dark-brown rhizome in *B. tulda* and abundant rooting (81.67%, *P* < 0.05, *F* = 15.46) for *M. baccifera* within 30 days were achieved. The established protocol and the validation of the reported species at the molecular level will be of help to stakeholders in edible bamboo trade to conserve gene-pool and increase productivity.

## 1. Introduction

The demand for bamboo is on the rise in Asian countries for their utility in handicraft industry, construction, paper making, fishery, and human consumption [1, 2]. Edible bamboos have been identified and grouped based on the total cyanogen content [3]. Such highly demanded edible bamboo with substantial nutritional attributes are* Dendrocalamus hamiltonii *[4–6],* Chimonobambusa callosa*,* Bambusa tulda*, and* Melocanna baccifera* [3].* M. baccifera* is sparsely found in the valleys of North Eastern states of India such as Manipur, Sikkim, Arunachal Pradesh, and Mizoram [7]. The market prices of fermented bamboo shoots for* B. tulda* and other species in India are at the range of Rs. 40 (*≈*US $0.66) to Rs. 50 (*≈*US $0.83) per kg [8], and they cost more than US $1.20 when canned or fried. Due to intense shifting cultivation and the use of fire for clearing the forest,* M. baccifera* is now restricted to the hilly regions and facing extinction.

There is no* in vitro* protocol for* B. tulda* reporting rhizogenesis herein described, and additionally, there is no report for micropropagation of* M. baccifera*. Thus, there is need to develop a new protocol for rhizome induction which forms buds that develops into new culms or shoots easily. Shoot regeneration and rooting are key steps for* in vitro* micropropagation. Recent advances in tissue culture explored the combined effects of silicon and sodium chloride to promote adventitious shoot regeneration in* Ajuga multiflora* [10]. Nonetheless, protocols based on zygotic embryos (i.e., explant from seed tissue) for woody bamboo plantlet regeneration have been developed [11–15]. Firstly, the limitation with zygotic embryos protocols in bamboo plantlets regeneration is that a given protocol is species specific and, consequently, does not often apply to other genera. Secondly, the long and irregular bamboo flowering cycle [16, 17], as well as low seed viability and scarcity in bamboo seeds [18, 19], often limits zygotic embryo protocols for bamboo plantlet regeneration. Therefore, new accurate protocols are required for successful propagation of several species since nodal explants are readily available.

*B. tulda* and* M. baccifera* are sympodial species which produce nutritive shoots [3, 20]. Protocols for plantlets regeneration were developed for* B. tulda* through seeds [11] and nodal explants [21], without achieving rhizogenesis. Till date, there is no protocol for plantlets regeneration for* M. baccifera*. Plantlets regeneration protocols in bamboo generally suffer from poor taxonomic identification and clonal fidelity for the reported species. Because of these setbacks, protocols reported on several bamboo species are frequently not reproducible. In this study, we first used trnL-F intergenic spacer to accurately ascertain the taxonomic placement of the studied species. Secondly, we developed an effective protocol for* in vitro* shoot proliferation from the nodal buds and optimized rooting of* B. tulda* and* M. baccifera*.

## 2. Material and Methods

### 2.1. Plant Material and Phylogenetic Placement

*Bambusa tulda* and* Melocanna baccifera* were identified by the authorities of the Botanical Survey of India (BSI), Kolkata. The lateral branches of the authenticated specimen were collected from culms of* B. tulda* and* M. baccifera* which lies 23°47′–25°41′ NL and 92°58′–94°47′ EL from the Forest Department of Imphal District, Manipur, India, during the month of July 2011. Genomic DNA was isolated as described [22]. The quantity and quality of DNA were checked on a nanodrop spectrophotometer (BioSpec-nano, Shimadzu, Japan) and 0.8% w/v agarose gel electrophoresis, respectively. The trnL-F intergenic spacer was amplified as previously described [23] and sequenced. Sequences were analysed and assigned to molecular species based on 98–100% sequence similarity in the GenBank as accessions: JX507132 (*Bambusa tulda*) and JX507133 (*Melocanna baccifera*), respectively. Sequences were aligned using Muscle program [24] and best substitution model was determined based on Akaike Information Criterion, corrected (AICc), and Bayesian Information Criterion (BIC). Phylogenetic analysis was performed using the maximum likelihood method in MEGA6 software [25].

### 2.2. Explants and Surface Sterilization

The leaf sheaths covering the nodal segments containing axillary buds (1.5–2 cm) were carefully removed and wiped with 70% (v/v) ethanol using sterilized cotton. The explants were surface sterilized in 0.1% (v/v) solution of mercuric chloride (HgCl_2_) for 15 min and washed 4 times, with each washing step lasting 5 min. The sterilized nodal segments were cultured in liquid Murashige-Skoog (MS) medium supplemented with 100 mg/L myo-inositol (Sigma, Saint Louis, MO, USA) and 30 g/L sucrose. It is worth noting that the pH of the medium was adjusted to 5.7 ± 0.1 with 1 N NaOH or 1 N HCl before autoclaving at 121°C and 117.68 kPa for 20 min. For bud breaking, the MS medium was supplemented with 6-benzylaminopurine (BAP) at 10 levels (1, 2, 3, 4, 5, 6, 7, 8, 9, and 10 mg/L).

### 2.3. Shoots Multiplication

Proliferated axillary shoots of 3–5 shoot clusters were excised and transferred to MS medium supplemented either with (1) BAP at 5 levels (1, 2, 3, 4, and 5 mg/L), (2) 6 mg/L kinetin (Kn) singly, or (3) combination of Kn at levels (1, 2, and 4 mg/L) with optimum concentration of 3 mg/L of BAP. It is worth mentioning that the choice of 6 mg/L of Kn at one level was based on preliminary finding where best effect was observed (data not shown). Shoot multiplication was performed using 3–5 axillary proliferated shoots excised from the clusters of shoots. Multiplied shoots in liquid cultures were supported by sterile filter paper bridges. Regularly, the shoots were transferred at interval of 10 days under sterile conditions to fresh medium in order to prevent phenolic oxidation that can trigger the shoots to become yellowish. The multiplied shoots on each culture medium were counted after 45 days to evaluate the multiplication rate of the nodal explants.

### 2.4. Rooting Process

We used 5 levels (1, 2, 3, 4, and 5%) of sucrose in half-strength MS medium to determine the optimum concentration that induces rooting. Furthermore, rooting was achieved by aseptically transferring multiplied shoots to a half-strength MS medium prepared with 3% sucrose (i.e., optimum concentration) and supplemented with different combinations of growth regulators as follows. For* B. tulda*, we used (1) 3 mg/L of indole-3-butyric acid (IBA), (2) 3 mg/L of IBA with 10 mg/L of coumarin, and (3) 3 mg/L of IBA, 3 mg/L of indole-3-acetic acid (IAA), and 10 mg/L of coumarin. For* M. baccifera*, we used (1) 3 mg/L of IBA, (2) 3 mg/L of IBA with 10 mg/L of coumarin, and (3) 3 mg/L of IBA, 0.05 mg/L of BAP, and 10 mg/L of coumarin. All cultures in the experiment were incubated in a growth chamber at 25 ± 1°C with a 16 h photoperiod at light intensity of 45 *μ*mol/m^2^/sec photosynthetic photon flux (PPF) provided by cool white fluorescent tubes (TLD Cool White 40W, Phillips, India). Rooting percentage and average number of days for rooting were recorded. Following rooting, MS medium was removed and the plantlets were hardened in a bottle jar of 76 mm height × 60 mm diameter and 143 mL cap containing autoclaved soil. The soil was made of 4 : 1 (% w/w) rice-straw-vermin compose and sand. The plantlets were cultured for 25 days. Acclimatization was achieved at 30 ± 2°C and at 84% humidity in the greenhouse.

### 2.5. Clonal Fidelity Test

We checked clonal fidelity by sequencing the trnL-F intergenic spacer of* in vitro* raised plantlets and compared the sequences with the mother explants.

### 2.6. Statistical Analysis

The experiments were performed in replicates for a total of 3 biological repeats carried out at separate times. All data on shoot regeneration and* in vitro* rooting were analysed using one-way ANOVA associated with Tukey's* post hoc* test at *P* ≤ 0.05. Statistical analysis was performed in IBM SPSS statistical software v.19.0.

## 3. Results and Discussion

### 3.1. Phylogenetic Placement of Species

**Figure 1:**
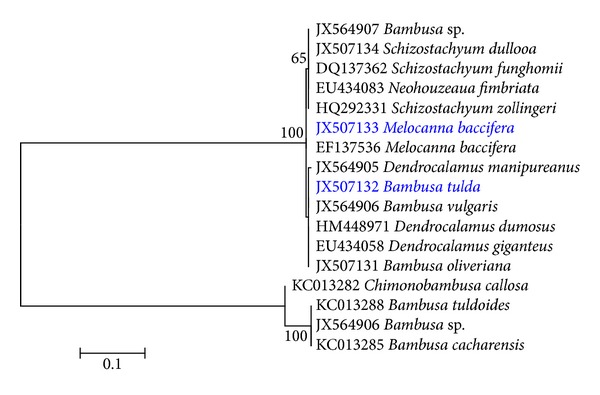
Molecular phylogenetic analysis by maximum likelihood method based on the T93 substitution model [9]. AIC is 1551.28, BIC is 1763.70, the highest log likelihood is −741.59, and bootstrap values ≥50% from 1000 iterations are shown. The taxa we developed* in vitro* plantlets regeneration based on nodal explants are highlighted in blue.

Based on trnL-F intergenic spacer sequence similarity test between* B. tulda*,* M. baccifera*, and closely related species, the overall mean distance between taxa was 0.54. A total of 167 patterns were found out of a total of 1040 sites, and 832 sites were without polymorphism (80.00%). The maximum likelihood (ML) inference authenticated the taxonomic placement of* Bambusa tulda* and* Melocanna baccifera* with respect to their closely related taxa (Figure 1). The ML inference confirmed the studied species were different. Failure to accurately identify bamboo species prior to propagation can lead to misleading reports on developed protocols. Motivated by recurring failure to achieve plantlets regeneration for* B. tulda* based on previously reported nodal protocol [21], we suspected the problem could probably be due to factors like species identity, the physiological state of mother explant, the quality of growth regulators used, and so forth. As reviewed in Mudoi et al. [16], poorly identified nodal clones, somatic clones, or the mother explant compromises the fidelity of protocols. Considering the species specificity of protocols in* in vitro* bamboo plantlets regeneration, we suggest that protocols should be validated at the DNA level for the identity of the explants so as to limit the number of factors that might affect reproducibility. There was no significant difference in the sequences of trnL-F intergenic spacer between mother explants and* in vitro* raised plantlets (data not shown). Thus, validating clonal fidelity is based on the developed protocol.

### 3.2. Culture Initiation and* In Vitro* Axillary Bud Break

**Figure 2:**
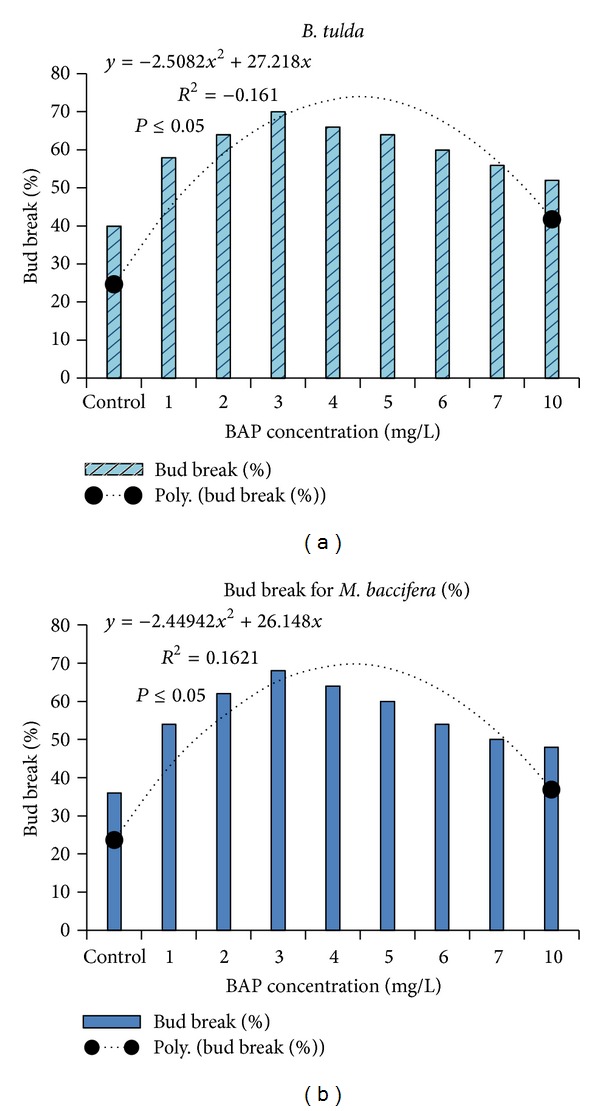
Effectiveness of BAP on bud break in MS medium revealed 3 mg/L is the optimum concentration. (a)* Bambusa tulda*. (b)* Melocanna baccifera*.

The explants of* B. tulda* and* M. baccifera* were established successfully on the modified MS medium with low rate of contamination. Previous work on micropropagation of* B. tulda* from nodal explants had encountered problems in culture initiation due to fungal contamination [21]. In this protocol, we minimized the level of contamination by avoiding the use of water for cleaning the lateral branches at the initial step of surface sterilization. Additionally, it is shown that supplementing MS medium with NaCl and silicon significantly enhances the activities of antioxidant enzymes [10] and equally curbs contaminations encountered in tissue culture. Axillary proliferation took place after 15 days of bud break for* B. tulda* and* M. baccifera*. The highest frequency of bud break was observed at the optimum concentration of 3 mg/L BAP for* B. tulda* and* M. baccifera.* This was hallmarked by the production of 1–3 shoots as a result of axillary bud break for* B. tulda* and* M. baccifera* (Figure 2).

**Figure 3:**
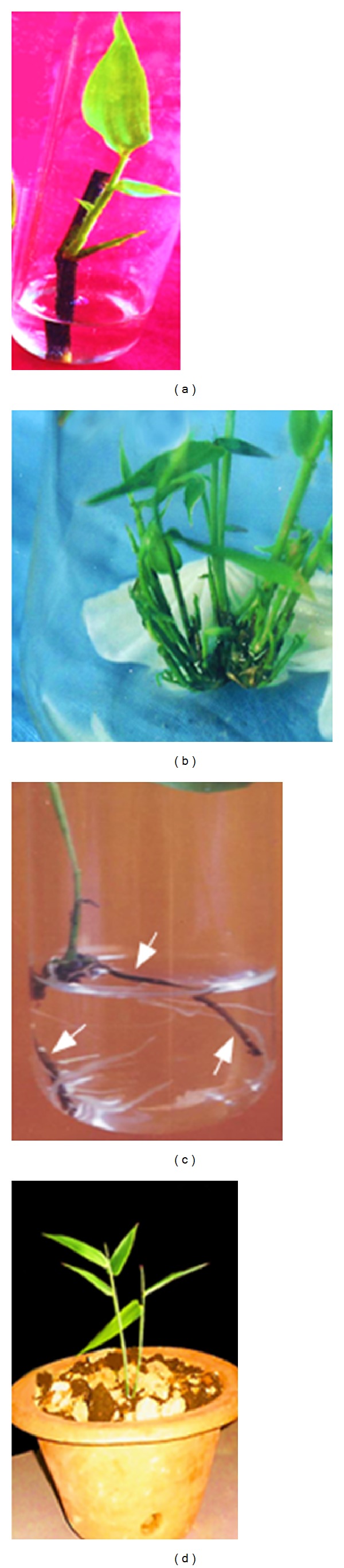
Micropropagation of* Bambusa tulda*. (a) Node with axillary bud breaks in liquid MS medium. (b) Shoot multiplication in 2 mg/L Kn + 3 mg/L BAP. (c) Formed rhizome (tagged with arrows) produced multiple profuse whitish roots in 0.5 MS medium supplemented with 3 mg/L IBA + 3 mg/L IAA + 10 mg/L coumarin + 3% sucrose. (d) Plantlets established in the soil under greenhouse conditions.

**Figure 4:**
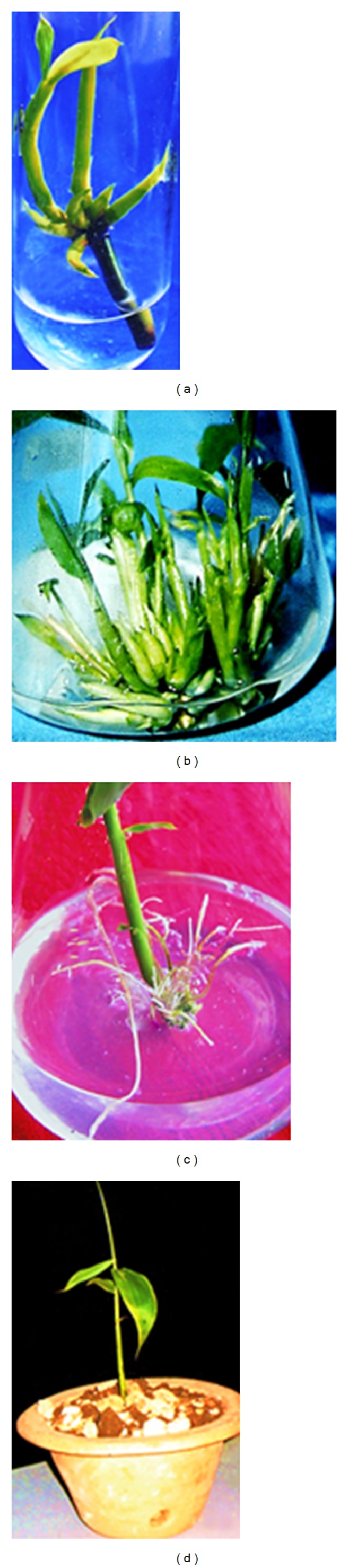
Micropropagation of* M. baccifera*. (a) Node with axillary bud breaks in liquid MS medium. (b) Shoot multiplication in 2 mg/L Kn + 3 mg/L BAP. (c) Rooting in 0.5 MS medium supplemented with 3 mg/L IBA + 10 mg/L coumarin + 0.05 mg/L BAP + 3% sucrose. (d) Plantlets established in the soil under greenhouse conditions.

As shown (Figure 2), above or below 3 mg/L of BAP, a low rate of bud break was recorded.* In vitro* bud break was low in the basal medium for* B. tulda* and* M. baccifera*. Although we tested other cytokinin such as Kn, only BAP efficiently promoted bud break at all tested concentrations. In previous studies, BAP was successfully used in bud breaking in plant species like* Arundinaria callosa*,* B. vulgaris*, and* Melocanna bambusoides* [26–28] and in* Origanum* species [29, 30]. Previous studies on* B. tulda* did not explore the effect of BAP on axillary bud break [21]. Herein, rapid bud breaking of axillary explants dormancy for* M. baccifera* and* B. tulda* was achieved at 3 mg/L of BAP in 10 and 15 days, respectively. Details of the bud break (Figures 2, 3(a), and 4(a)), shoot multiplication (Figures 3(b) and 4(b)), and* in vitro* rooting (Figures 3(c) and 4(c)) are depicted for* B. tulda* and* M. baccifera*.

### 3.3. Effects of BAP and Kn on Shoot Multiplication

In this study, it was observed that increase in concentration of BAP (2–4 mg/L) enhanced the rate of shoot multiplication. Nonetheless, above 5 mg/L of BAP, sharp decline in the shoot multiplication rate was observed (Figures 3(b) and 4(b)). BAP has been observed to be the most effective plant growth regulator for woody bamboo shoot* in vitro* multiplication [28, 31, 32]. In our study, the highest shoot multiplication rate per explant was 5.50 (*P* < 0.05, *F* = 3.18) in* B. tulda* under the influence of 3 mg/L BAP and 2 mg/L Kn (Table 1). Also, the maximum shoot multiplication rate per explant was 5.83 (*P* < 0.05, *F* = 3.85) in* M. baccifera* under the effect of 3 mg/L BAP and 2 mg/L Kn (Table 2). Comparatively, a single dose effect of Kn on* B. tulda* and* M. baccifera* did not increase the rate of shoot multiplication. Interestingly, the combined effect of BAP and Kn increased shoot multiplication rate as well as the quality of shoots in* B. tulda* and* M. baccifera* (Figures 3(b) and 4(b)). In this regard, the effective combined concentration was 2 mg/L of Kn and 3 mg/L of BAP which produced 17.67 and 18.17 of average shoots per explant in* B. tulda* and* M. baccifera*, respectively.

To achieve optimal shoot multiplication, other authors have combined synthetic auxin (such as 1-naphthalene acetic acid, NAA), natural auxin (such as IBA) with cytokinin (such as BAP) in culture medium [4, 27, 33]. In some cases, combination of BAP with Kn enhanced the rate of shoot multiplication [14, 34]. In the present investigation, combination of BAP and Kn produced a synergistic effect leading to an increase in shoot multiplication as well as the texture of shoots which turned deep green in both* B. tulda* and* M. baccifera* (Figures 3(b) and 4(b)). In contrast, Mishra et al. [21] achieved shoots multiplication of* B. tulda* by combining BAP, IAA, and glutamine.

### 3.4. Rooting Process and Rhizome Formation

**Figure 5:**
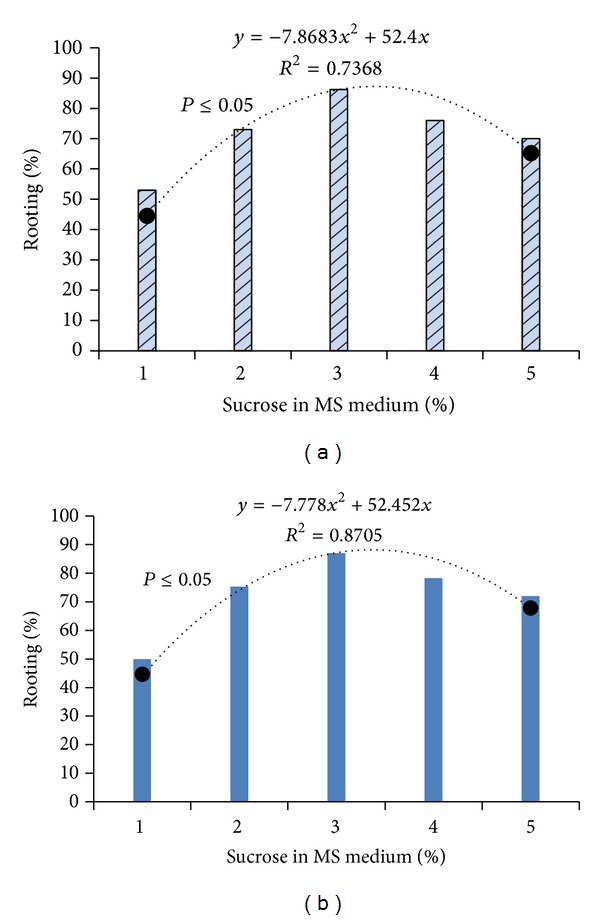
Effect of sucrose on rooting in MS medium revealed 3% is the optimum concentration. (a)* Bambusa tulda.* (b)* Melocanna baccifera*.

The shoot clusters of 1-2 cm transferred to a half-strength MS medium fortified with different plant growth regulators were successfully rooted. As shown by the regression analysis (Figure 5), varied concentrations of sucrose in combination with 3 mg/L IBA + 10 mg/L of coumarin influenced the rooting percentage in* B. tulda* and* M. baccifera* (Figure 5, Table 3). Optimum rooting was obtained with 3% of sucrose. Primarily, at 3% sucrose we obtained increased rooting in* B. tulda* and* M. baccifera*. It was observed that 3 mg/L IBA + 3% sucrose produced moderate rooting of 45.00% at an average rate of 76 days in* B. tulda*. Adding 10 mg/L of coumarin in IBA supplemented medium increased rooting efficiency from 45.00% to 77.00% (Table 3). Interestingly, rhizome formation occurred in the culture medium supplemented with 3 mg/L of IBA + 10 mg/L of coumarin + 3% sucrose for* B. tulda*. Importantly, the highest rate of roots regenerated from the nodes of rhizome stood at 86.70% within 25 days in MS medium supplemented with 3 mg/L of IBA + 3 mg/L of IAA + 10 mg/L of coumarin + 3% sucrose.* B. tulda* produced dark-brown rhizome from which profuse whitish roots emerged (Figure 3(c)). Rhizogenesis is hard to achieve in* in vitro* micropropagation of woody bamboo. Nonetheless, similar rhizome morphology was described in* Dendrocalamus asper* and* D. membranaceus* in the presence of 3% sucrose in MS medium [35]. It is worth noting that a unique dose of 3 mg/L of IBA + 3% sucrose produced low level rooting (55.00%, *P* < 0.05, *F* = 2.35) for* M. baccifera* (Table 3). To achieve optimal rooting, we combined 3 mg/L IBA + 10 mg/L coumarin + 0.05 mg/L BAP + 3% sucrose. This combination produced 81.67% rooting within 30 days (Table 3). No rhizome formation occurred in all the growth regulators combinations for* M. baccifera* culture (Table 3). In this study, we exploited the potentials of sucrose, BAP, coumarin, and IBA to root* B. tulda* and* M. baccifera*. Previous findings demonstrated that induction of* in vitro* rooting from shoot clusters in bamboos required auxins treatment alone or combined either with BAP or coumarin [14, 21, 36–38]. As shown in this study, coumarin was required in combination with IBA and BAP for promoting root induction in* B. tulda* and* M. baccifera*. Coumarin is a phenolic compound which may act synergistically with IBA to enhance endogenous liberation of IAA in explant during rooting process [39]. The freed endogenous IAA in turn favours roots initiation as earlier described [39]. Plantlets were successfully acclimatized (Figures 3(d) and 4(d)) under greenhouse conditions at a survival rate of 81.81% and 70.31% for* B. tulda* and* M. baccifera*, respectively.

## 4. Conclusion

In these established protocols, 3 mg/L BAP was found to be effective for bud break and shoot multiplication of* B. tulda* and* M. baccifera*. Rhizogenesis in bamboo micropropagation is difficult to achieve and has not been reported previously in* B. tulda* protocols [11, 21]. The developed protocol successfully produced rhizome in half-strength MS medium supplemented with 3 mg/L IBA, 10 mg/L coumarin, and 3% sucrose for* B. tulda* for the first time. This study provides an effective protocol for micropropagation of* B. tulda* and* M. baccifera* from the nodal segments of the field grown culm. This protocol shall be of help to stakeholders in edible bamboo trade to conserve gene pool and increase productivity.

## Figures and Tables

**Table 1:** Effect of BAP, Kn, and Kn + BAP in MS medium on shoot multiplication of *Bambusa tulda*.

Growth hormones	Concentration (mg/L)	Average shoots produced per explant	Multiplication rate per explant
BAP	1	8.33^a^ ± 1.82	2.50^ab^ ± 0.67
	2	14.00^abc^ ± 1.80	4.33^ab^ ± 0.67
	3	16.83^c^ ± 0.48	5.33^b^ ± 0.21
	4	16.50^bc^ ± 0.92	5.17^ab^ ± 0.31
	5	11.83^abc^ ± 1.94	3.50^ab^ ± 0.67

Kn	6	7.50^a^ ± 2.31	2.17^a^ ± 0.83

Kn + BAP	1 + 3	13.83^abc^ ± 2.81	4.17^ab^ ± 1.01
	2 + 3	17.67^c^ ± 0.56	5.50^b^ ± 0.22
	4 + 3	13.67^abc^ ± 2.32	4.17^ab^ ± 0.87

Mean of 10 replicates ± S.E., scored after 4 weeks in culture medium. Within each column, values followed by the same letter are not significantly different according to Tukey's test at *P* < 0.05.

**Table 2:** Effect of BAP, Kn, and Kn + BAP in MS medium on shoot multiplication of *Melocanna baccifera*.

Growth hormones	Concentration (mg/L)	Average shoots produced per explant	Multiplication rate per explant
BAP	1	8.50^a^ ± 1.80	2.67^a^ ± 0.61
	2	14.17^ab^ ± 1.96	4.50^ab^ ± 0.67
	3	17.00^b^ ± 0.44	5.33^b^ ± 0.21
	4	16.67^b^ ± 0.95	5.0^b^ ± 0.44
	5	12.00^ab^ ± 2.14	3.67^ab^ ± 0.76

Kn	6	15.83^b^ ± 1.28	4.83^ab^ ± 0.48

Kn + BAP	1 + 3	17.17^b^ ± 0.87	5.50^b^ ± 0.34
	2 + 3	18.17^b^ ± 0.31	5.83^b^ ± 0.17
	4 + 3	15.83^b^ ± 1.14	4.83^ab^ ± 0.48

Mean of 10 replicates ± S.E., scored after 4 weeks in culture medium. Within each column, values followed by the same letter are not significantly different according to Tukey's test at *P* < 0.05.

**Table 3:** Optimized rooting conditions in 0.5 strength MS medium in the presence of multiple growth hormones at 3 mg/L sucrose.

Plant growth regulators	Concentration (mg/L)	% rooting	Mean days
	*Bambusa tulda *		
IBA	3	45.00^a^ ± 4.94	76.00^ab^ ± 4.03
IBA + coumarin	3 + 10	77.00^b^ ± 7.0	51.67^a^ ± 7.83
IBA + IAA + coumarin	3 + 3 + 10	86.67^b^ ± 3.33	25.00^ab^ ± 4.83

	*Melocanna baccifera *		
IBA	3	55.00^a^ ± 7.56	40.83^ab^ ± 2.71
IBA + coumarin	3 + 10	75.00^b^ ± 8.57	36.67^ab^ ± 3.42
IBA + BAP + coumarin	3 + 0.05 + 10	81.67^b^ ± 6.54	30.00^ab^ ± 2.07

Mean of 10 replicates ± S.E., scored after 4 weeks in culture medium. Within each column, values followed by the same letter are not significantly different according to Tukey's test at *P* < 0.05.
